# Research and Discussion on the Relationships between Noise-Induced Hearing Loss and ATP2B2 Gene Polymorphism

**DOI:** 10.1155/2019/5048943

**Published:** 2019-12-01

**Authors:** Suhao Zhang, Enmin Ding, Haoyang Yin, Hengdong Zhang, Baoli Zhu

**Affiliations:** ^1^School of Public Health, Nantong University, Nantong, Jiangsu Province, China; ^2^Institute of Occupational Disease Prevention, Jiangsu Provincial Center for Disease Prevention and Control, Nanjing, Jiangsu Province, China; ^3^Center for Global Health, China International Cooperation Center for Environment and Human Health, School of Public Health, Nanjing Medical University, Nanjing, Jiangsu Province, China

## Abstract

Long-term and continuous noise exposure can result in noise-induced hearing loss (NIHL), which is a worldwide problem resulting from the interaction of environmental and genetic factors. The ATP2B2 gene polymorphism can destroy cochlear hair cells and increase the risk of NIHL. A case-control study of 760 Chinese textile workers was conducted to investigate the relationship between *ATP2B2* polymorphisms and NIHL susceptibility. Venous blood was collected and questionnaires were conducted by professional physicians. A case group and a control group which were typed by individuals' pure-tone audiometry test results were set. Three polymorphism sites of *ATP2B2* were genotyped by using the PCR technique. Analysis results revealed that the C allele of rs3209637 (95%CI = 1.08–2.58, odds ratio (OR) = 1.67, *P* = 0.027) was a dangerous factor and could add to risks of NIHL in the Chinese employees. The data of stratified analysis revealed that individuals who are exposed to noise > 95 dB with the rs3209637 C genotype have a higher susceptibility to NIHL (OR = 1.34, 95%CI = 1.07–1.68). Multifactor dimensionality reduction analysis revealed that the interaction between rs14154 and rs3209637 is linked to increased NIHL risk, and for the interaction among rs14154, smoking and drinking had the same function (OR = 1.54 and 1.77, 95%CI = 1.15–2.07, 1.33–2.37, and *P* = 0.0037 and *P* < 0.0001, respectively). Our results suggest that genetic polymorphism rs3209637 C within *ATP2B2* is a risk factor for NIHL among Chinese employees and rs3209637 C could be a potential biomarker for NIHL patients.

## 1. Background

Noise is a common occupational hazard in modern society which can cause permanent and irreversible damage to the human hearing system. NIHL is a primary occupational disorder worldwide and the second most common type of sensorineural hearing impairment with the second highest incidence [1]. According to reports, there are 10 million people suffering from NIHL in the USA and the group of NIHL patients in China has expanded 77.8% in three years (2010-2012). With the increasing number of NIHL patients, NIHL has caused serious harm to workers' health and socioeconomic conditions and becomes an important aspect of occupational prevention and control.

From previous studies, it can be concluded that NIHL is a multifactor disease influenced by external environmental factors and internal genetic factors [2]. Both physical factors, such as noise, chemicals, and heat, and personal behaviors could change the susceptibility of NIHL, such as smoking, drinking, and medical factors [3–8]. Eliminating interference from external environmental factors, individuals always showed different degrees of hearing loss under the same level of noise exposure, indicating that genetic susceptibility is a significant catalyst in the development of NIHL [9, 10]. Sliwinska-Kowalska and Pawelczyk [1] found that gene-knockout mice have expressed more susceptibility to noise than their wild-type littermates and proved that genetic polymorphisms contribute to occurrence of NIHL. Many genetic experiments had demonstrated that single nucleotide polymorphisms (SNPs) in the *DFNA5*, *FOXO3*, *heat shock protein 70*, and *EYA4* genes are genetic risk factors for NIHL in humans and can promote or reduce the occurrence of NIHL with the participation of external factors [10–12].

ATPase, calcium-transporting, plasma membrane 2 (*ATP2B2*) belongs to the *ATP2B* gene family and lies on human chromosome 3p25.3 and encodes Ca^2+^ pump *PMCA2* which is scattered around the plasma membrane and functions to pump Ca^2+^ out of the cell with ATP. High-level expression of *ATP2B2* in cochlear outer hair cells maintains the homeostasis of intracellular calcium [13], and when individuals were exposed to noise, the expression level of *ATP2B2* would change and lead to the changes of calcium concentration in hair cells and extracellular calcium concentration in the inner ear [14]. *ATP2B2* can be an early warning gene for NIHL, and low expression of *ATP2B2* would lead to neurodevelopmental defects of auditory systems and result in hearing loss [15, 16].

Although many animal studies were performed to reveal the associations between *ATP2B2* and the auditory system, the associations within the population were rarely explored [17, 18]. Documenting previous experiments, we speculated that the polymorphisms in *ATP2B2* could be one of the risk factors for NIHL. A case-control study was designed and conformed to analyze the potential link between *ATP2B2* SNPs (rs1719571, rs14154, and rs3209637) and genetic susceptibility to NIHL, and the mechanism by which ATP2B2 leads to NIHL is discussed in this article.

## 2. Materials and Methods

### 2.1. Research Objectives

This study had achieved authorization from the Research Ethics Committee of Jiangsu Provincial Center for Disease Prevention and Control (JSCDC), and all the studies were conducted in accordance with correlative standards and rules. Each participant signed the informed consent before the studies. The industrial employees in a Chinese textile manufactory were recruited and underwent medical health examinations every year as executed by the JSCDC. Occupational health test projects principally consisted of physiological and biochemical examinations, routine physical examination, and pure-tone audiometry (PTA). In health tests, personal drug history, smoking status, drinking status, and commonly used medicines were investigated by questionnaire. The participants in the following situations were removed: people had diseases which could influence hearing thresholds, such as otitis media, diabetes, and nephropathy, and participants had taken or were taking ototoxic drugs. Afterwards, 760 individuals confirmed to our requirements and participated in the studies.

### 2.2. PTA and NIHL Evaluation

Each participant avoided noise exposure for at least 12 hours and was tested for the PTA experiment in a soundproof room by using an audiometer (Madsen, Taastrup, Denmark).

### 2.3. Individual Noise Exposure Measurement

The random sampling method is adopted to select workers to wear a personal noise dosimeter. The Quest NoisePro DL multifunctional individual noise dosimeter (Quest, USA) was used to measure the individual noise exposure dose during work hours. Then, an 8-hour equivalent continuous sound level (A) was calculated.

### 2.4. Defining Normal and Hearing Loss Subjects

Participants were detected for audiometry and divided into the hearing loss group and the normal hearing group according to diagnostic criteria for occupational noise-induced deafness in China (GBZ 49-2007). In this research, workers who continued to be exposed to at least 85 dB noise during an 8-hour working day were defined as suffering from occupational noise exposure. Participants with bilateral threshold deviations of more than 25 dB at high and low frequencies were classified as the hearing loss groups. Oppositely, participants with bilateral threshold deviations of less than 25 dB at high and low frequencies were classified as the normal hearing groups. We determined patient population and then matched the control groups based on sex, age, and degrees of noise exposure. 380 NIHL cases and 380 controls from the individuals met our requirements and were selected finally.

### 2.5. DNA Extraction

Three milliliters of peripheral blood were collected in EDTA-containing anticoagulant tubes for DNA extraction and genotyping. DNA was extracted from participants' blood samples by the QIAcube HT and QIAamp 96DNA QIAcube HT Kit under guidance of the manufacturers' instructions. The abstracted DNA was stored up at -20°C for future use.

### 2.6. SNP Selection and Genotyping

*ATP2B2* was selected as the target SNPs based on the 1000 Genomes Project and previous literature results. The standards for identifying SNPs included (a) minor allele frequency (MAF) of the Chinese Han population (CHB) > 0.10 and (b) linkage disequilibrium *r*^2^ value >0.8. Haploview 4.2 software was adopted to measure the LD patterns of SNPs with *r*-squared value and distinguished three SNPs (rs1719571, rs14154, and rs3209637) in *ATP2B2*. Combining with a review of literature from PubMed and Web of Science, we found that the rs1719571, rs14154, and rs3209637 of the *ATP2B2* gene were studied frequently and thus selected for genotyping.

Using predesigned commercial genotyping assays, polymorphic genotypes were selected with ABI company TaqMan SNP genotyping assays. Samples were genotyped by an ABI 7900 HT real-time PCR system. Then, the outcomes were analyzed by the ABI 7900 system sequence detection software.

### 2.7. Statistical Analysis

SPSS 23.0 software (IBM, USA) was adopted to analyze the experimental data. The goodness-of-fit *χ*^2^ tests were used for the Hardy-Weinberg equilibrium rule of the SNPs in *ATP2B2* genes in the control group. Classification variables are described in percentages, and continuous variables are described in the mean ± SD. Conditional logistic regression models were adjusted for age, sex, smoking, and drinking and used to calculate the odds ratios (ORs) and 95% confidence intervals (95% CI) for genotypes [12]. The interaction between gene and dangerous factors was analyzed by multifactor dimensionality reduction (MDR). We adopted *P* < 0.05 as the standard for measuring statistical significance.

## 3. Results

### 3.1. Demographic Characteristics of Study Individuals and Hardy-Weinberg Tests of ATP2B2 Genotype

The common features, such as age, sex, smoking status, drinking status, working time with noise, noise level, and high-frequency hearing thresholds of the NIHL groups and controls, are shown in Table 1. Comparing the general characteristics between the NIHL groups and control groups, no significant differences were found between different populations (*P* > 0.05) except the hearing threshold. The case groups exposed to high-frequency noise have a higher average hearing threshold (36.74 ± 10.23) than the control groups exposed to low-frequency noise (13.66 ± 4.20; *P* < 0.001). The results of three chosen SNPs and Hardy-Weinberg tests are shown in Table 2, and *χ*^2^ tests indicated every chosen SNP was in Hardy-Weinberg equilibrium (*P* > 0.05).

### 3.2. Association between ATP2B2 SNPs and Risks of NIHL

The relationship between *ATP2B2* and the NIHL risk was analyzed by multivariate analysis. Three *ATP2B2* SNPs were chosen to genotype in the 760 noise-exposed employees (380 NIHL patients and 380 controls). The genotype and allele distributions of rs1719571, rs14154, and rs3209637 under recessive, dominant, codominant, and allelic models are shown in Table 3. Table 3 showed significant variances of genotype frequencies of rs3209637 existed obviously between the cases and controls under the codominant model (*P* = 0.027). People with a higher proportion of the ATP2B2 C allele are more susceptible to NIHL with reference to the ATP2B2 T allele (*P* = 0.033). Moreover, the rs3209637 CC was significantly related to enhancive NIHL risks under the recessive model (*P* = 0.007). Logistic regression analysis adjusted for sex, age, smoking, and drinking revealed that the rs3209637 CC genotype increased NIHL hazard (OR = 1.66, 95%CI = 1.16–2.37). Under the allelic model, the rs3209637 C (95%CI = 1.01–1.52, OR = 1.24) allele revealed an enhancive risk for NIHL (*P* = 0.045).

### 3.3. Interactions between rs1719571, rs14154, and rs3209637 Polymorphisms and Cumulative Noise Exposure

The analysis results of interactions between the SNPs and cumulative noise exposure were analyzed under an allelic model and are exhibited in Table 4. 95 dB was the cumulative average noise exposure and chosen as the standard. There was an obviously significant variance between the case and control groups with rs3209637 C when individuals' cumulative noise exposure was >95 dB (95%CI = 1.07–1.68, OR = 1.34, and *P* = 0.015).

### 3.4. Interactions among SNPs and Dangerous Factors

**Figure 1:**
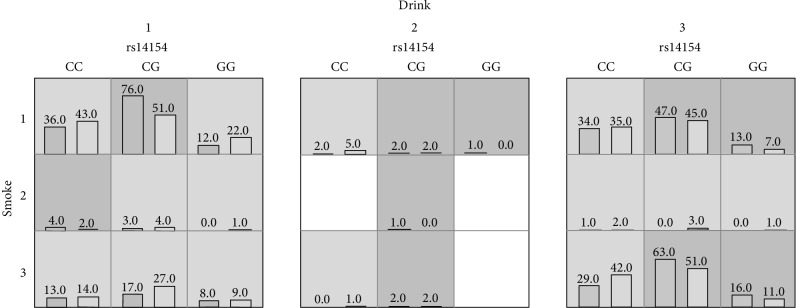
Best-fit model gained by the analysis of MDR. The implications of the bars and background color in each multifactor cell are as follows. The left bars represent the sum of scores in the case and the right represents the control. High risk cells are expressed by the black shadow if the ratio of the number of cases to the number of controls exceeds the preset value *T*, as low risk cells by light shadow if not more than the threshold, and empty cells with no shadow which means no cases and controls. The multifactor cells labeled as “high risk” or “low risk” are then used to assess the classification and predication accuracy, thus identifying the best model in the subsequent steps (drink 1: now; drink 2: ever; drink 3: never; smoke 1: now; smoke 2: ever; smoke 3: never).

The consequences of the multifactor dimensionality analysis of interaction among three SNPs and other factors are expressed in Table 5. The analysis revealed that for the interactions between rs14154 and rs3209637 and among rs14154, smoking and drinking were associated with increased NIHL risk (OR = 1.54 and 1.77, *P* = 0.0037 and *P* < 0.0001, correspondingly). Diagrams of the best-fit model are shown in Figure 1.

## 4. Discussion

SNP, the mutation of single nucleotides, is an ordinary phenomenon in the human genome and there are 15 million SNPs in all humans [19]. Although the SNPs distribute in humans widely, SNPs do not distribute homogeneously and most SNPs are in noncoding areas of the genome. Currently, there are many methods to detect the SNPs, such as allele-specific PCR. Except for the allele-specific PCR, scientists also adopt numerous ways to perform researches, for instance, cleaved amplified polymorphic sequence (CAPS), single-strand conformational polymorphism (SSCP), and denaturing gradient gel electrophoresis (DGGE) [14].

According to the literature review, although there are many animal experiments researching the surface relationship between ATP2B2 and NIHL and studies on the Chinese population were rare, human mutation in ATP2B2 which described a young deaf subject presented a variant in the ATP2B2 gene associated with a variant in the Cadherin 23 gene [20]. In this study, we adopted TaqMan genotyping to analyze the three selected *ATP2B2* SNPs (rs1719571, rs14154, and rs3209637) in 380 NIHL cases and 380 controls and found statistically significant associations between the three SNPs and NIHL hazard. Demographic characteristic calculations for the population ensured homogeneity between the two groups. The significant difference between the case group and the control group showed that the noise would change the high-frequency hearing threshold (*P* < 0.001). We also found that the rs3209637 C genotype of *ATP2B2* (OR = 1.24, 95%CI = 1.01–1.52, *P* = 0.045) was significantly relative with a higher NIHL risk in which the result is a powerful evidence for our hypothesis that *ATP2B2* polymorphisms could increase the NIHL susceptibility in the Chinese employees. The stratified analysis of SNPs in the allelic model showed NIHL risk of individuals increased when subjects' cumulative noise exposure is >95 dB. This result showed the addition effect between cumulative noise exposure and rs3209637 C genotype of ATP2B2. Finally, the interaction between rs14154 and rs3209637 increased the risk of NIHL. The rs3209637 also interacted with smoking and drinking and led to the enhanced hazard of NIHL. Our study is an association research demonstrating that the *ATP2B2* genes would enhance the NIHL risk among the Chinese population.

Till now, the mechanism of NIHL is still unclear, but numerous scientists widely accept that the DNA impairment in cochlear hair cells seriously damages the growth of NIHL, especially when individuals are exposed to massive noise [21]. Many scientists also put forward that the apoptosis and necrosis of inner ear cells which were caused by the oxidative stress or metabolic products and structural damage which directly affected the cochlear structure may be the most possible etiopathogenesis of NIHL [21–23].

Some knockout mouse research demonstrated that the *ATP2B2* gene encodes *PMCA2* [17]. *PMCA2* can pump Ca^2+^ to the endolymph without the natural activator calmodulin [24], and mutant *PMCA2* expressed in CHO cells led to calcium extrusion impairment [16]. *ATP2B2* was induced by exogenous Zn^2+^ to change its expression and caused cell death by disrupting calcium homeostasis [25]. Disruption of calcium homeostasis was an important incentive to many diseases, like breast cancers, autism, deafness, and ASD [13, 16, 24, 26, 27].

The scientists considered that *ATP2B2* could be the key role during the development of NIHL and found that the absence of *ATP2B2* could decrease *PMCA2* concentration, affect the calcium homeostasis in hair cells, and finally cause defects of auditory systems [15, 16]. *ATP2b* has four subtypes: *PMCA1*, *PMCA2*, *PMCA3*, and *PMCA4*. These four subtypes are highly similar and consist of 75-80% amino acids and are distributed in different areas of the human body. *PMCA1* and *PMCA4* are widely distributed in the whole body, and *PMCA2* mainly exists in the sensory cells of the inner ear. Many animal studies were conducted to research the association between *PMCA2* and NIHL. *PMCA2* can regulate the intracellular Ca^2+^ concentration in hair cells of static fibers. High concentration of Ca^2+^ in hair cells could produce toxicity and damage hair cells. As a result, the physiological functions of static fibers are damaged, the damaged static fibers would influence the human auditory system. *PMCA2* acts as a beneficial gene by pumping excessive Ca^2+^ out of hair cells to maintain the homeostasis of Ca^2+^ concentration in the auditory system. The genotype rs3209637 is located in 3′-UTR within *ATP2B2* and could change the conserved miR-137 binding site at the end of the human *ATP2B2* distal 3′UTR. mir-137 has an important role in the human auditory system, which may demonstrate the association between rs3209637 polymorphism and NIHL [28].

In the current study, smoking and drinking were both risk factors of NIHL. The interaction among smoking, drinking, and rs3209637 had an association with NIHL. Mohammad et al. [29] had suggested smoking is a contributing factor to hearing loss. In Iran, Mofateh et al. [30] also found smoking had an additive role in hearing loss interacting with massive noise exposure in high frequency. The same situation happened in Sarawak and 20 packs of cigarettes per year were found as the risk factor [29]. Eliminating the effects of noise, alcohol could cause hearing loss and the influence was particularly significant at 1000 Hz [31]. An experiment in Hong Kong found the association between drinking and NIHL and confirmed the conclusion that drinking would increase the risk of NIHL [32]. Our results were consistent with the previous experimental results and confirmed drinking and smoking were both risk factors. Our studies expanded the sample size and discussed the interaction between risk factors and genotypes. The experimental results show that there were additive effects between individual factors and SNPs, which is consistent with the conclusions of previous experiments.

There are still many shortcomings in our experiments. First of all, our study's sample size may be larger than sizes in previous studies. But the effectiveness of statistical tests in our study was inadequate to discuss the small biological effects of individual SNPs. Thus, the consequent targets are performing more massive sample size researches, cohort studies, and functional experiments in the future. We could research the function of the *ATP2B2* polymorphisms on NIHL hazard through these experiments. Secondly, the candidates in our study were mostly recruited from Chinese employees and had regional restrictions. The researches had racial limitations, and experimental results were more applicable to Chinese employees and cannot be extended to other populations.

## 5. Conclusion

In general, the rs3209637 C genotype of *ATP2B2* may lead to a greatly increased incidence of NIHL. The analysis also demonstrates that *ATP2B2* SNPs (rs1719571, rs14154, and rs3209637) have a great effect in NIHL and these SNPs could be researched and developed to play a great role in the prevention of NIHL by functioning as a biomarker.

## Figures and Tables

**Table 1:** Demographic characteristics of study subjects.

Variables	Case group (*n* = 380)		Control group (*n* = 380)		*P*
	*n*	%	*n*	%	
Age (years)					
Mean ± SD	39.82 ± 6.59			40.09 ± 6.32	0.567^a^
Sex					
Male	358	94.2	355	93.4	0.652^b^
Female	22	5.8	25	6.6	
Tobacco use					
Now	223	58.7	210	55.3	0.501^b^
Ever	9	2.4	13	3.4	
Never	148	38.9	157	41.3	
Alcohol consumption					
Now	169	44.5	173	45.5	0.836^b^
Ever	8	2.1	10	2.6	
Never	203	53.4	197	51.8	
Work time with noise (years)					
Mean ± SD	17.35 ± 7.60		16.94 ± 7.16		0.452^a^
Expose level with noise (dB)					
Mean ± SD	89.52 ± 7.26		90.09 ± 6.98		0.269^a^
High-frequency hearing threshold (dB)					
Mean ± SD	36.74 ± 10.23		13.66 ± 4.20		**<0.001** ^c^
≤25	0	0.0	380	100.0	
>25	380	100.0	0	0.0	

^a^Students' *t*-test. ^b^Two-sided *χ*^2^ test. ^c^Fisher's exact test.

**Table 2:** General information of selected SNPs and Hardy-Weinberg test.

SNP	Alleles	Chromosome	Functional consequence	MAF		*P* for HWE^b^
				Control	Database^a^	
rs1719571	A/G	3:10327496	3′UTR	0.357	0.360	0.903
rs14154	C/G	3:10326429	3′UTR	0.378	0.383	0.751
rs3209637	C/T	3:10327264	3′UTR	0.463	0.465	0.959

^a^Data from NCBI dbSNP. ^b^*P* value of Hardy-Weinberg test.

**Table 3:** Distribution of three polymorphisms and the association with NIHL.

Genetic models	Genotypes	Case group		Control group		*P* ^a^	Adjusted OR (95% CI)^b^
		*n* = 380	%	*n* = 380	%		
rs1719571							
Codominant	AA	141	37.1	161	42.4	0.332	1.00 (ref.)
	AG	183	48.2	167	43.9		1.25 (0.92-1.70)
	GG	56	14.7	52	13.7		1.26 (0.81-1.96)
Dominant	AA	141	37.1	161	42.4	0.138	1.00 (ref.)
	GG+AG	239	62.9	219	57.6		1.25 (0.93-1.67)
Recessive	AG+AA	324	85.3	328	86.3	0.678	1.00 (ref.)
	GG	56	14.7	52	13.7		1.11 (0.74-1.68)
Alleles	A	465	61.2	489	64.3	0.203	1.00 (ref.)
	G	295	38.8	271	35.7		1.15 (0.94-1.42)

rs14154							
Codominant	CC	119	31.3	144	37.9	0.129	1.00 (ref.)
	CG	211	55.5	185	48.7		**1.39 (1.02-1.90)**
	GG	50	13.2	51	13.4		1.21 (0.76-1.92)
Dominant	CC	119	31.3	144	37.9	0.057	1.00 (ref.)
	CG+GG	261	68.7	236	62.1		**1.35 (1.00-1.83)**
Recessive	CG+CC	330	86.8	329	86.6	0.915	1.00 (ref.)
	GG	50	13.2	51	13.4		0.99 (0.65-1.51)
Alleles	C	449	59.1	473	62.2	0.208	1.00 (ref.)
	G	311	40.9	287	37.8		1.15 (0.94-1.42)

rs3209637							
Codominant	TT	83	21.8	92	24.2	**0.027**	1.00 (ref.)
	CT	203	53.4	224	58.9		1.01 (0.71-1.44)
	CC	94	24.7	64	16.8		**1.67 (1.08-2.58)**
Dominant	TT	83	21.8	92	24.2	0.438	1.00 (ref.)
	CC+CT	297	78.2	288	75.8		1.15 (0.82-1.62)
Recessive	CT+TT	286	75.3	316	83.2	**0.007**	1.00 (ref.)
	CC	94	24.7	64	16.8		**1.66 (1.16-2.37)**
Alleles	T	369	48.6	408	53.7	**0.045**	1.00 (ref.)
	C	391	51.4	352	46.3		**1.24 (1.01-1.52)**

^a^Two-sided *χ*^2^ test. ^b^Adjusted for age, sex, tobacco use, and alcohol consumption in the logistic regression model.

**Table 4:** Stratified analysis of SNPs in the allelic model.

SNPs	Group	Alleles	Cumulative noise exposure (dBA)	
			≤95	>95
rs1719571	Case group	G	67	228
		A	109	356
	Control group	G	43	228
		A	91	398
	*P* ^a^		0.276	0.373
	Adjusted OR (95% CI)^b^		1.32 (0.82-2.13)	1.12 (0.89-1.42)

rs14154	Case group	G	70	241
		C	106	343
	Control group	G	47	240
		C	87	386
	*P* ^a^		0.398	0.298
	Adjusted OR (95% CI)^b^		1.24 (0.77-1.98)	1.14 (0.91-1.44)

rs3209637	Case group	C	88	303
		T	88	281
	Control group	C	71	281
		T	63	345
	*P* ^a^		0.602	**0.015**
	Adjusted OR (95% CI)^b^		0.90 (0.57-1.42)	**1.34 (1.07-1.68)**

^a^Two-sided *χ*^2^ test. ^b^Adjusted for age, sex, tobacco use, and alcohol consumption in logistic regression model.

**Table 5:** Analysis of the interaction of the 3 selected SNPs by MDR.

Best model	Training balanced accuracy	Testing balanced accuracy	Cross-validation consistency	*P*	OR (95% CI)
rs3209637	0.5408	0.5105	7/10	**0.0073**	**1.62 (1.14-2.32)**
rs14154∗rs3209637	0.5541	0.5158	6/10	**0.0037**	**1.54 (1.15-2.07)**
rs14154∗smoke∗drink	0.5756	0.4711	5/10	**<0.0001**	**1.77 (1.33-2.37)**

## Data Availability

The data used to support the findings of this study are available from the corresponding author upon request.
